# Supplementary material for: The short- and long-term temporal relation between falls and concern about falling in older adults without a recent history of falling
Source: PLoS One. 2021 Jul 9;16(7):e0253374. doi: 10.1371/journal.pone.0253374 (PMC8270453; doi:10.1371/journal.pone.0253374)
Supplement: S1 Table — (PDF) [file pone.0253374.s001.pdf]

Supplementary material - Temporal relation between falls and concern about falling in older adults without history of falling

S1 Table. Relation between falling and concern about falling over long-term and short-term intervals. This table shows the crude regression coefficients (Beta, or change in FES-I score) or Odds Ratio (OR) of the long term, determined with regression models, and the short term, determined with linear mixed models, effects of the main independent variables (before the arrows) on the dependent variables (after the arrow). The table includes results both when all falls are analyzed ("All falls") or when falls are only considered if they resulted in any injury ("Injurious falls"). * indicates statistical significance at alpha = 0.050

	All falls<br>beta/OR [95% CI]	p-value	Injurious falls<br>beta/OR [95% CI]	p-value
**Fall → FES-I (beta)**				
Long-term (1 year)	0.91 [-0.10, 1.92]	0.077	0.62 [-0.44, 1.67]	0.250
Short-term (1 month)	1.49 [0.75, 2.24]	<0.001*	2.60 [1.57, 3.63]	<0.001*
**FES-I → Fall (OR)**				
Long-term (1 year)	1.09 [0.98, 1.23]	0.143	1.08 [0.97, 1.21]	0.154
Short-term (1 month)	1.03 [0.95, 1.10]	0.457	1.06 [0.96, 1.16]	0.181
