# Supplementary material for: The short- and long-term temporal relation between falls and concern about falling in older adults without a recent history of falling
Source: PLoS One. 2021 Jul 9;16(7):e0253374. doi: 10.1371/journal.pone.0253374 (PMC8270453; doi:10.1371/journal.pone.0253374)
Supplement: S2 Table — (PDF) [file pone.0253374.s002.pdf]

Supplementary material - Temporal relation between falls and concern about falling in older adults without history of falling

S2 Table. Participant characteristics of both participants with and without a recent history of falling. All values are means (standard deviation) assessed at baseline unless otherwise noted. IQR = Inter quartile range. Changes in walking duration and FES-I scores from baseline assessment to one-year later are presented, positive values represent increases, negative values represent decreases. [1] information on six participants was missing. [2] information on two participants was missing. [3] information on one participant was missing.

	**Without a recent history of falling (N=118)**		**With a recent history of falling (N=114)**	
Age, years	71.4 (5.3)		71.7 (5.8)	
Female, n (%)	82 (69.5)		79 (69.3)	
Body height, cm	169.0 (8.3)		169 (7.9)	
Body weight, kg	73.8 (12.6)		73.1 (13.8)	
Baseline Quickscreen, n (%)	[1]		[2]	
7% fall risk	45 (38.1)		4 (3.5)	
13% fall risk	60 (50.8)		75 (65.8)	
27% fall risk	4 (3.4)		21 (18.4)	
49% fall risk	3 (2.5)		12 (10.5)	
Combined hand grip force, kg	60.4 (16.7)		57.1 (15.0)	
Baseline walking duration, min/day	84.6 (31.4)		82.0 (30.3)	
One-year change in walking duration, min/day	-0.2 (19.9)		1.86 (21.5)	
Uses walking aid, n (%)	3 (2.5)[3]		8 (7.0)	
Highest achieved education, n (%)				
Higher education	96 (81.4)		94 (82.5)	
Lower secondary education	19 (16.1)		16 (14.0)	
Primary education	3 (2.5)		4 (3.5)	
MMSE score, median [IQR]	28 [28, 29]		29 [28, 30]	
Self-reported comorbidities, n (%)				
Diabetes	5 (4.2)		10 (8.8)	
High blood pressure	35 (29.7)		28 (24.6)[3]	
Low blood pressure	6 (5.1)		6 (5.3)	
Cerebral infarction	5 (4.2)		3 (2.6)	
Myocardial infarction	9 (7.6)		1 (0.9)	
Thyroid condition	6 (5.1)		7 (6.1)	
Asthma or Chronic Obstructive Pulmonary Disease	9 (7.6)		7 (6.1)	
Pain in joints	50 (42.4)		57 (50)	
Osteoporosis	15 (12.7)[3]		18 (15.8)[3]	
Time between completion of monthly follow-up questionnaires, median [IQR], days	30 [28, 35]		30 [28, 35]	
Baseline FES-I score, median [IQR]	18 [17, 21]		20 [18, 23]	
One-year change in FES-I score, median [IQR]	0 [-1, 1]		0 [-2, 1]	
Number of falls experienced during one-year follow-up, n (%)	All falls	Injurious falls	All falls	Injurious falls
None	58 (49.2)	78 (66.1)	47 (41.2)	73 (64.0)
One	30 (25.4)	31 (26.3)	34 (29.8)	21 (18.4)
Two or more	30 (25.4)	9 (7.6)	33 (28.9)	20 (17.5)
