# Supplementary material for: The short- and long-term temporal relation between falls and concern about falling in older adults without a recent history of falling
Source: PLoS One. 2021 Jul 9;16(7):e0253374. doi: 10.1371/journal.pone.0253374 (PMC8270453; doi:10.1371/journal.pone.0253374)
Supplement: S3 Table — (PDF) [file pone.0253374.s003.pdf]

S3 Table. Relation between falling and concern about falling over long-term and short-term intervals in participants with a recent history of falling. This table shows the regression coefficients (Beta, or change in FES-I score) or Odds Ratio (OR) of the long term, determined with regression models, and the short term, determined with linear mixed models, effects of the main independent variables (before the arrows) on the dependent variables (after the arrow). The table includes results from analyses with all falls and only Injurious falls. # 95% CI could not be determined as the model resulted in a singular fit. * indicates statistical significance at alpha = 0.050; The effects are adjusted for age [1], gender [2] and walking duration per day [3] as described in the methods section.

	**All falls beta/OR [95% CI]**	**p-value**	**Injurious falls beta/OR [95% CI]**	**p-value**
**Fall → FES-I (beta)**				
Long-term (1 year)	0.15 [-1.00, 1.30][3]	0.796	0.05 [-1.15, 1.26][1,3]	0.929
Short-term (1 month)	0.54 [-0.43, 1.51]	0.276	0.05 [-1.47, 1.57][1,2,3]	0.878
**FES-I → Fall (OR)**				
Long-term (1 year)	0.99 [0.91, 1.08][2,3]	0.827	1.01 [0.93, 1.09][2]	0.889
Short-term (1 month)	0.99 [0.92, 1.06][2,3]	0.868	0.96 [--, --][#,1,2,3]	0.548
