# Supplementary material for: The short- and long-term temporal relation between falls and concern about falling in older adults without a recent history of falling
Source: PLoS One. 2021 Jul 9;16(7):e0253374. doi: 10.1371/journal.pone.0253374 (PMC8270453; doi:10.1371/journal.pone.0253374)
Supplement: Attachment — Submitted filename: Response to Reviewers.docx [file pone.0253374.s005.docx]

1. Please ensure that your manuscript meets PLOS ONE's style requirements, including those for file naming. The PLOS ONE style templates can be found at

<https://journals.plos.org/plosone/s/file?id=wjVg/PLOSOne_formatting_sample_main_body.pdf> and

<https://journals.plos.org/plosone/s/file?id=ba62/PLOSOne_formatting_sample_title_authors_affiliations.pdf>

2.

We note that you have indicated that data from this study are available upon request. PLOS only allows data to be available upon request if there are legal or ethical restrictions on sharing data publicly. For more information on unacceptable data access restrictions, please see <http://journals.plos.org/plosone/s/data-availability#loc-unacceptable-data-access-restrictions>.

In your revised cover letter, please address the following prompts:

a) If there are ethical or legal restrictions on sharing a de-identified data set, please explain them in detail (e.g., data contain potentially sensitive information, data are owned by a third-party organization, etc.) and who has imposed them (e.g., an ethics committee). Please also provide contact information for a data access committee, ethics committee, or other institutional body to which data requests may be sent.

b) If there are no restrictions, please upload the minimal anonymized data set necessary to replicate your study findings as either Supporting Information files or to a stable, public repository and provide us with the relevant URLs, DOIs, or accession numbers. For a list of acceptable repositories, please see <http://journals.plos.org/plosone/s/data-availability#loc-recommended-repositories>.

We will update your Data Availability statement on your behalf to reflect the information you provide.

3. Please amend the manuscript submission data (via Edit Submission) to include author O.G. Meijer.

[Note: HTML markup is below. Please do not edit.]

Reviewers' comments:

Reviewer's Responses to Questions

**Comments to the Author**

1. Is the manuscript technically sound, and do the data support the conclusions?

The manuscript must describe a technically sound piece of scientific research with data that supports the conclusions. Experiments must have been conducted rigorously, with appropriate controls, replication, and sample sizes. The conclusions must be drawn appropriately based on the data presented.

Reviewer #1: Yes

Reviewer #2: No

Reviewer #3: Yes

2. Has the statistical analysis been performed appropriately and rigorously?

Reviewer #1: Yes

Reviewer #2: No

Reviewer #3: Yes

3. Have the authors made all data underlying the findings in their manuscript fully available?

The [PLOS Data policy](http://www.plosone.org/static/policies.action#sharing) requires authors to make all data underlying the findings described in their manuscript fully available without restriction, with rare exception (please refer to the Data Availability Statement in the manuscript PDF file). The data should be provided as part of the manuscript or its supporting information, or deposited to a public repository. For example, in addition to summary statistics, the data points behind means, medians and variance measures should be available. If there are restrictions on publicly sharing data—e.g. participant privacy or use of data from a third party—those must be specified.

Reviewer #1: No

Reviewer #2: No

Reviewer #3: Yes

4. Is the manuscript presented in an intelligible fashion and written in standard English?

PLOS ONE does not copyedit accepted manuscripts, so the language in submitted articles must be clear, correct, and unambiguous. Any typographical or grammatical errors should be corrected at revision, so please note any specific errors here.

Reviewer #1: Yes

Reviewer #2: Yes

Reviewer #3: Yes

5. Review Comments to the Author

Please use the space provided to explain your answers to the questions above. You may also include additional comments for the author, including concerns about dual publication, research ethics, or publication ethics. (Please upload your review as an attachment if it exceeds 20,000 characters)

Reviewer #1: This is a very interesting – and well-designed – study that explores both the short- and long-term relationship between concerns about falling (assed via the FES-I) and falls, both injurious and non-injurious. While previous research (e.g., Friedman et al.) has explored this relationship, there is need to isolate this exploration both temporally (ie, short- vs long-term) and in non-fallers. This research question has high levels of relevance for the clinical managing of individuals presenting concerns about falling. I commend the authors for conducting a clinically and scientifically important piece of research. I do, however, have a number of issues – some large, some small – which I feel need to be addressed before I can fully recommend the research for publication. Nonetheless, overall, this is a solid piece of work.

*We would like to thank the reviewer very much for his/her thorough review of our manuscript. Dealing with the suggestions and questions has increased the quality of our manuscript.*

• Introduction – Line 49 – Should this not be “…we studied community-dwelling older adults without a RECENT history of falls”? As you only studied whether participants fell in the 12 months prior to your research, and not beyond this point. (So you may have had a participant who fell multiple times in the past few years, and simply did not fall in the 12 months prior to participation.) I would also recommend changing the abstract and title to reflect that the history of falling refers to a recent history of falling.

*We agree that it is unlikely or actually impossible to find people who have never fallen and we only assessed the one year prior to baseline. We have changed the title and all lines in the manuscript that mentioned “without a history of falling” to “without a recent history of falling”.*

• On this point, I know that it is standard practice to assign participants to a faller/non-faller group based on the no. of falls over the past 12 months. But in my clinical experience, falls from beyond a 12-month period tend to also influence concern about falling and behavior that may either increase or decrease risk of falls – particularly if the fall was particularly serious/injurious. For example, an injurious fall 24 months ago may lead to a perpetual state of fear about falling; but due to experiencing a fall, the individual now attends physiotherapist-led exercise classes which improve balance and decrease the risk of future falls. Similarly, someone may have experienced a serious/injurious fall 2 years prior that drastically increased concerns about falling to the extent that any future falls leaves concerns unchanged (given the already high level of concerns). It might be worth adding this as a potential limitation.

*The reviewer addresses an important limitation that cannot be controlled for given the study design. We have addressed the limitation in the discussion section as follows:*

Line 449: “Despite the strength of our study to exclude people with a recent history of falling it is possible that a fall before the year prior to the baseline assessment has had a lasting influence on concern about falling. Such an effect may have influenced our results as we did not assess fall history for more than one year before baseline assessment. However, since we found no evidence of long-lasting effects of falls on concern about falling, it is unlikely that fall history from the period of earlier than one year before the start of our study to have influenced our findings substantially.”

• I feel that the Introduction is lacking in any description of how concern about concerns about falling may influence falls risk either indirectly (e.g., activity avoidance and deconditioning) or directly (e.g., adoption of ‘high-risk’ behaviors). For the indirect point, it would be worth referencing the paper by Hadjistavropoulos et al. 2011 (DOI: 10.1177/0898264310378039). For the direct point, it would be worth describing the work by Brown et al. (DOI: 10.1007/s00221-003-1468-7), Delbaere et al. (DOI: 10.1093/gerona/gln014) and Ellmers et al. (DOI: <https://doi.org/10.1093/gerona/glz176>). The reader needs to know how concerns of falling may influence fall risk.

*We thank the reviewer for the suggestion of relevant studies. We have included the following paragraph to the introduction to provide a background for the hypothesis that concern about falling increases risk of falling:*

Line 56: “Pathways through which concern about falling may affect the risk of falling have been described previously. Attention allocation while walking may be altered in response to concern about falling (5) and hence threatening objects in the surrounding receive more visual and cognitive attention (6). This adaptation seems to give more time to anticipate obstacles, but becomes counterproductive in more complex environments with multiple obstacles, especially while dual-tasking (7, 8), and may lead to trips and falls (9, 10). Furthermore, concern about falling can indirectly lead to falling through activity restriction and thereby induced physical decline (11, 12). It should be noted that it may take time for physical decline to become so severe that it increases fall risk.”

*The following references have been added:*

“[5] Gage WH, Sleik RJ, Polych MA, McKenzie NC, Brown LA. The allocation of attention during locomotion is altered by anxiety. Experimental brain research. 2003;150(3):385-94.

[6] Young WR, Mark Williams A. How fear of falling can increase fall-risk in older adults: applying psychological theory to practical observations. Gait Posture. 2015;41(1):7-12.

[7] Ellmers TJ, Cocks AJ, Kal EC, Young WR. Conscious Movement Processing, Fall-Related Anxiety, and the Visuomotor Control of Locomotion in Older Adults. The Journals of Gerontology: Series B. 2020;75(9):1911-20.

[8] Ellmers TJ, Young WR. Conscious motor control impairs attentional processing efficiency during precision stepping. Gait & posture. 2018;63:58-62.

[9] Young WR, Hollands MA. Newly Acquired Fear of Falling Leads to Altered Eye Movement Patterns and Reduced Stepping Safety: A Case Study. PloS one. 2012;7(11):e49765.

[10] Young WR, Wing AM, Hollands MA. Influences of state anxiety on gaze behavior and stepping accuracy in older adults during adaptive locomotion. The journals of gerontology Series B, Psychological sciences and social sciences. 2012;67(1):43-51.

[11] Delbaere K, Crombez G, Vanderstraeten G, Willems T, Cambier D. Fear-related avoidance of activities, falls and physical frailty. A prospective community-based cohort study. Age and ageing. 2004;33(4):368-73.

[12] Hadjistavropoulos T, Delbaere K, Fitzgerald TD. Reconceptualizing the role of fear of falling and balance confidence in fall risk. Journal of aging and health. 2011;23(1):3-23.”

• How was your sample size calculated?

*Sample size for the VIBE study was calculated based on a chi-square test to identify if people who over or under-estimated their physical ability were more at risk of falling. Alpha was set at 0.025, beta at 0.80, effect size was estimated at 0.13 with 3 degrees of freedom. A total sample size of 337 participants was found to be sufficient with these parameters. The VIBE cohort ended with 287 participants included for baseline assessments and 255 participants at the follow-up assessment 12 months after baseline. We did not perform a power analysis prior to the analysis described in the current study, instead we used all available data from the VIBE cohort.*

• How valid is using minutes walking per day recorded at the very start of data collection (ie, before any falls) as a covariate for the relationship between variables throughout the study? Traditional models of fear of falling would suggest that following a fall, individuals will reduce their physical activity – meaning that any physical activity recording from pre-fall may no longer be valid. Is there a more appropriate physical functioning covariate that you could include instead? If possible, one that is likely to be more stable over time and less affected by the fall itself. Maybe functional balance (Berg balance), strength or even gait speed?

*The reviewer notes that the amount of physical activity a participant participates in may change after having experienced a fall and that physical activity may be a less appropriate physical functioning covariate than, for instance, the Berg balance scale.
First, we did not include physical activity as a covariate to control for physical functioning. Instead, we included it to control for exposure to balance threats and to control for experience through which concern about falling may be updated. This has been better described as follows under covariates and we have updated it with a reference:*

*Line 146: “*The amount of walking activity may also affect our findings, as more active people may be more exposed to situations in which they are at risk of falling, but more active people may also have a better estimate of their physical ability (19) and this estimate may affect the level of concern a person has about falling. We therefore included walking duration per day to control for exposure to balance threats and to control for experience through which concern about falling may be updated.*”*

*“[19] Sakurai R, Fujiwara Y, Suzuki H, Ogawa S, Higuchi T, Imanaka K. Changes in self-estimated step-over ability among older adults: A 3-year follow-up study. The Journals of Gerontology: Series B. 2020.”*

*Second, we agree with the reviewer that physical activity may change after a fall. While writing a response to reviewer 2’s comments we found that we had forgotten an essential part in our methods section, which may also be relevant to this comment of reviewer 1. For the one-month, i.e., short-term, period, we only analyzed data up to and including the subsequent questionnaire assessment after an individual’s first fall. Hence, although the level of physical activity may have changed after a fall, it will not affect the results of our one-month, i.e., short-term, analysis. Furthermore, on the long term, we found no significant within participant change in walking duration from baseline to one-year follow-up and have included the follow-up change in physical activity levels in Table 1. At baseline, the mean (sd) time was 84.6 (31.0) minutes, while at follow-up the mean (sd) time was 82.5 (27.6) minutes, with an average change per participant of -0.2 (19.9) minutes. The following has been added to the manuscript:*

*Methods:*

*Line 152: “*Walking duration per day was determined from inertial sensor data collected directly following the baseline and one-year follow-up assessments for seven times 24 consecutive hours.*”*

*And*

Line 184: “Statistical analyses were aimed at determining whether concern about falling at the start of a short-term (one month) or the long-term (12-month) period is predictive for falling within such a period and whether falling within such a period is predictive of having an increase in concern about falling at the end of that period compared to the start of that period. All analyses were performed twice ~~We assessed both long term (one year) and short term (one month) relations between falls and concern about falling and all analyses were performed twice~~: first with all reported falls and then with injurious falls only. For the short-term (one-month), interval, we only analyzed data up to and including the assessment at the end of a one-month period during which a person had fallen for the first time. Any subsequent assessments were excluded from the analysis as that person would no longer be a person without a recent history of falling at that time.*”*

*Results – table 1:*

| Baseline walking duration, min/day | 84.6 (31.4) |
| --- | --- |
| One-year change in walking duration, min/day | -0.2 (19.9) |

*Third, the reviewer also suggested to include an alternative physical functioning or performance measure such as the berg balance scale. As pointed out above, we did not include physical functioning as a covariate. Furthermore, we are hesitant to include more covariates because of lack of power. A general rule of thumb regarding predictors in (logistic) regression models is to include 1 predictor per 10 events (Peduzzi and colleagues 1996, DOI:* *10.1016/S0895-4356(96)00236-3). Since there are a total of 40 injurious falls in our study, adding more than 4 predictors is not advised as it may tend to make the results less valid. The aim of this study was to compare the interrelation between falls and concern about falling. Therefore, both sides of relation should be tested with models that were corrected for the same variables and hence also the linear regression models should not be corrected for by more than 4 covariates. Therefore, we did not include a physical functioning measure as a covariate.*

• The statistical analysis section is very well described, thank you for putting so much focus into this. The Results section were very clear as well. Excellent job.

*We thank the reviewer for these kind words.*

• Table 1 – would it be possible to report FES values at the end of data collection? The baseline scores are very low (much lower than what we normally observe following clinical recommendation for falls prevention services). I am interested to see how these scores changed over the year.

*We agree that the FES-I values found in the VIBE cohort are relatively low in comparison with several other studies. We have added the change of scores to Table 1 (see next comment).*

• Do you have any other participant demographic data to report here? Berg scale? Timed up and go? Cognition? I also think it would be helpful to report the range for all variables.

*We agree and have added the following to Table 1. Variables in red are new, updates to numerical values (see comment about missing values of covariates by reviewer 2). These are not accentuated but can be reviewed in the tracked changes version of the manuscript. A measure of variance is given for all variables (either standard deviation, inter quartile range or percentage).*

|  | **N=118** | |
| --- | --- | --- |
| Age, years | 71.4 (5.3) | |
| Female, n (%) | 82 (69.5) | |
| Body height, cm | 169.0 (8.3) | |
| Body weight, kg | 73.8 (12.6) | |
| Baseline Quickscreen fall risk score, n (%)^1^ |  | |
| Very low fall risk | 45 (38.1) | |
| Low fall risk | 60 (50.8) | |
| Moderate fall risk | 4 (3.4) | |
| High fall risk | 3 (2.5) | |
| Combined hand grip strength, kg | 60.4 (16.7) | |
| Baseline daily walking duration, min/day | 84.6 (31.4) | |
| One-year change in daily walking duration, min/day | -0.2 (19.9) | |
| Use of walking aid, n (%) | 3 (2.5)^2^ | |
| Highest achieved education, n (%) | | |
| Higher education | 96 (81.4) | |
| Lower secondary education | 19 (16.1) | |
| Primary education | 3 (2.5) | |
| MMSE score, median [IQR] | 28 [28, 29] | |
| Self-reported comorbidities, n (%) |  | |
| Diabetes | 5 (4.2) | |
| High blood pressure | 35 (29.7) | |
| Low blood pressure | 6 (5.1) | |
| Cerebral infarction | 5 (4.2) | |
| Myocardial infarction | 9 (7.6) | |
| Thyroid condition | 6 (5.1) | |
| Asthma or Chronic Obstructive Pulmonary Disease | 9 (7.6) | |
| Pain in joints | 50 (42.4) | |
| Osteoporosis | 15 (12.7)^1^ | |
| Time between completion of monthly follow-up questionnaires, median [IQR], days | 30 [28, 35] | |
| Baseline FES-I score, median [IQR] | 18 [17, 21] |  |
| One-year change in FES-I score, median [IQR] | 0 [-1, 1] |  |
| Number of falls experienced during the one-year study period ~~follow-up~~, n (%) | All falls | Injurious falls |
| None | 58 (49.2) | 78 (66.1) |
| One | 30 (25.4) | 31 (26.3) |
| Two or more | 30 (25.4) | 9 (7.6) |

*We have also added the following paragraph to the method section:*

*Line 171: “Population descriptives*

*The Quickscreen fall risk assessment tool was used to indicate fall risk at the start of the study (23). The Quickscreen consists of the assessment of physical performance, fall history and use of medication. Based on the number of identified risk factors, participants were divided into one of four subgroups: very- low (0 or 1 risk factor), low (2 or 3 risk factors), moderate (4 risk factors) or high (5 or more risk factors) fall risk, as described in Tiedemann and colleagues 2010.
Participants’ handgrip strength was assessed with a dynamometer (24). They were instructed to squeeze the dynamometer as forceful as possible, twice with each hand, while standing and holding their arms stretched downwards by their side without touching their body. We calculated the mean peak force (in kilograms) from the hands and summed the highest scores of each hand.
Number of comorbidities, the use of walking aids and education were self-reported by participants.”*

• In previous work, Delbaere et al. (DOI: <https://doi.org/10.1136/bmj.c4165>) have shown that the relationship between concerns about falling and fall-risk may not be linear, and may instead be determined by physiological falls risk (which was not assessed or controlled in the present research). Can you please comment on how this may have affected your results?

*We agree with the reviewer that physiological capacity/fall risk may play an important role. We have changed the following to the discussion:*

Line 321: “~~We found that concern about falling is increased after one month, but not after one year, when a person falls in between assessments. This finding seems to be in contrast to~~ Our findings on the relations between falls and concern about falling in both directions appear to be in contrast with the studies by Delbaere and colleagues (13, 14). They ~~,who~~ showed that concern about falling did not develop differently in groups of people who had not fallen or had fallen (multiple times). ~~In our study, increased concern about falling after a fall was only observed in the one-month follow-up interval but not in the one-year follow-up interval . The follow-up interval in the study by Delbaere and colleagues (14) was three months. It is possible that the~~ Possibly, the short-term effects of a fall on concern about falling that we observed in one-month intervals was washed out within the three months~~’ time~~ intervals of Delbaere and colleagues (14) ~~but are visible over a one month interval~~. In the opposite direction of the relation, they showed that concern about falling was predictive of future falls (13) which is in contrast to our findings. These discrepancies between our study and the studies by Delbaere and colleagues (13,14) may be explained by the fact that we excluded people with a self-reported history of falling in the previous year, which we deem a strength of our study and necessary to accurately assess the relationship between falling and concern about falling.
First, the selection of participants without a recent history of falling is reflected in our FES-I scores, which indicate ~~Another explanation, possibly related to the difference in exclusion criteria concerning fall history, could be that the participants in the study by Delbaere and colleagues had an average FES-I score of 22.6 (SD 6.4) at baseline, indicating~~ that ~~their~~the participants of Delbaere and colleagues, including people with a recent fall history, were more concerned (~~average~~ mean FES-I 22.6 with SD 6.4 (14)) than our participants (~~average~~ mean FES-I 19.~~0~~ 2 with SD ~~4.0~~3.7). Perhaps, a proportion of their participants ~~were~~was already concerned about falling at baseline and did not become more concerned after a subsequent fall.~~. We found that concern about falling was not predictive of future falls, neither over a one-month period nor over a one-year period. This is in contrast to the findings of Delbaere and colleagues (13). In the previous paragraph, we already indicated that the population of Delbaere and colleagues (13, 14) was more concerned about falling at baseline than our population. Perhaps~~ Likewise, our population may have been too unconcerned about falling, and the range of FES-I scores too limited, for us to replicate their results. ~~This might raise questions about the representativeness of our study population. However, we do believe that our study population was a good representation of community dwelling older adults without a recent history of falling as these people are unlikely to have high concern about falling. It can therefore be questioned whether concern about falling can be considered as a primary cause for falls.~~

Second, our selected population consists mostly of participants with good physiological capacity as indicated by the high percentages of participants in the very low and low fall risk groups determined from the Quickscreen fall risk assessment. As suggested by Delbaere and colleages (13), the relationship between falling and concern about falling may be dependent on physiological fall risk, because concern about falling may increase the risk of trips and thereby falls. Hence, people with good physiological capacity may be better able to compensate for this increased risk than people with poor physiological capacity. This may be a reason why we did not find that concern about falling was predictive of falling in our population. We do believe that our study population was a good representation of community dwelling older adults without a recent history of falling as these people are unlikely to have high concern about falling.”

• I would be very interested to see how the results differ for the participants with a recent history of falling. Would it be possible to conduct these analyses and attach them as a supplementary appendix? If these results show similar to the previous studies by Friedman et al. and Delbaere et al., then you have strong evidence for the reason for the discrepancy between your study and this previous work – it is due to the influence of fallers, in this previous work.

*We deem the selection of people without recent fall history a major strength of our paper in the methods and results, and we do see added value in additional analyses on participants with a history of falling for comparison and explanation of discrepancies with literature. We therefore added these findings to the supplementary material and addressed these findings and comparisons in the discussion section:*

Line 364: “The question remains whether concern about falling can be considered as a primary cause for falls or whether it differs between people with or without a recent history of falling. Therefore, we performed the same analyses described in this manuscript on the participants with a recent history of falling (see S2 Table for population descriptives) and found no significant associations between falls and concern about falling, neither in either direction nor for the type of falls, injurious or all falls (S3 Table). Hence, participants without a recent history of falling had increased concern about falling over one month when they had fallen in between the start and end of that period (Table 2), but people with a recent history of falling did not (S2 Table). This may indicate that concern in participants with a recent history of falling was already elevated and was not further affected after a subsequent fall, conform the findings by Delbaere and colleagues (14). These comparisons further support our belief that self-reported history of falling may either bias findings concerning the relationship between falls and concern about falling, and that this relationship is different for people with or without a recent history of falling. Moreover, to evaluate the association between recent fall history and concern about falling, we performed linear regression analyses similarly to the long-term, i.e., one-year, models described in the method section. In one model recent fall history was associated with concern about falling at baseline and we compared its results with a second model in which falls recorded during the one-year monthly follow-up predicted concern about falling at the end of that follow-up (not adjusted for concern about falling at baseline). The results and comparison of these results indicated that history of falling was associated with high concern about falling at baseline (βfall= 1.79, 95% CI [0.64 , 2.93], points on the FES-I, p = 0.002), whereas number of falls during the one-year follow-up was not associated with concern about falling one year after baseline (βfall= 1.05, 95% CI [-0.28, 2.38], points on the FES-I, p = 0.123) and (βinjuriousfall= 0.86, 95% CI [-0.53, 2.25], points on the FES-I, p = 0.225). Recall bias may be an explanation for this finding as recall bias is likely to be more pronounced when the recall period is large, i.e., when asking about recent fall history retrospectively instead of assessing falls prospectively using a monthly follow-up. Concern about falling may possibly lead to recall bias such that people with high concern about falling recall more falls than people with low concern about falling. This recall bias stresses the need for prospective fall assessments using monthly telephone contact or fall diaries, such as we employed in our study, to validly record falls in older adults in future studies.”

• In your reference list, reference number 6 is blank?

*There appears to be a confusing lay-out issue that causes this “6.” to seem like a blank reference whereas it actually is the ending page number of the reference 14: ”…* *Age and ageing. 2010;39(2):210-6.”. the “6.” jumped to the next line as it did not fit on the line above it. This should not be confusing anymore after final lay-out has been applied.*

Reviewer #2: Overall
This research examined a cohort or elderly who had no falls within the past year, studying fear of falling and fall occurrence. However, a 1-year follow up after falls was not completed (1-year data was estimated from linear regression) and statistical analysis methods were not supported in the text (analysis did not seem to relate to the research question). Also some missing data was manufactured instead of removing the trial. Based on these deficiencies, I stopped reviewing after methods since I cannot trust the results.

*We value the time and effort the reviewer has invested in our manuscript. Based on his/her comments we believe that we have not been successful in clearly describing the methods used in this study. "(1-year data was estimated from linear regression)" (above) and " you cannot say that this is long term 1 year response to falling." (below) revealed to us that our text could be read as if we analyzed data through one year after a fall. Our "1-year follow-up" phrasing may have created that impression. We apologize. We determined whether FES-I scores changed (in covariation with other variables) from the beginning to the end of a month, and from the baseline/first assessment until the study stopped one year after the baseline assessment). We have replaced the "1-year follow-up" in our phrasing into "1-year after the baseline assessment". We thank the reviewer for pointing this out to us.*

Abstract:
• I am not aware of a controversy for falls and fear of falling, best to remove this wording

*We agree that this wording was poorly chosen given the limited amount of context of an abstract. We have replaced it as follows:*

Line 15: “The ~~interrelation~~ reciprocal relation between falling and concern about falling is complex and ~~a matter of controversy~~ not well understood.”

Data sharing
• The statement about 15 year data storage is common for institutions since it indicates that long term data storage is required for studies and then the media can be destroyed. If your statement that this clause negates any data sharing then PLOS One is liable for all data sharing that has had institutional ethics review (i.e., likely every study with humans published). I expect that the authors are incorrect and that they should share the data as per PLOS One guidelines.

*Unfortunately this could not be realized before resubmission but we will make	the data publically available before the manuscript is published.*

Introduction
• Line 64: what is meant by “the interrelations between falls and concern about falling”, what are interrelations in this context?

*We agree that the meaning of “interrelation” is somewhat ambiguous given the	limited context. We had meant to convey the notion that the relation between	falls and concern about falling is bi-directional, falls may influence concern and	concern may increase risk of falls. We have changed it to “*reciprocal relation*”.*

• Line 66: what is meant by “direction of relation”? is this a vector?

*The direction of relation refers to the fact that the relation may be reciprocal. Hence, falls may influence concern about falling and concern about falling may influence the number of falls a person experiences. By having changed the ambiguous term interrelation into “reciprocal relation”, we hope that the idea of two directions to the relation between falls and concern for falling is clear to future readers.*

Methods
• How many people fell multiple times? How did you handle analysis for time period after multiple falls?

*As presented in Table 1, 30 (24.8%) people fell twice or more often and 9 (7.4%) experienced two or more injurious falls.
The reviewer directed our attention to a piece of information that was missing from the manuscript. For the short term (i.e., one-month) assessments we only investigated the relationship between falls and concern about falling from one month to the next month for the first pair of months in between which a person had fallen. FES-I and fall data from months that follow this first pair of months are excluded from this part of the analysis.*

*We have included the following sentence in the method section:*
Line 190: “*. For the short-term (one-month), interval, we only analyzed data up to and including the assessment at the end of a one-month period during which a person had fallen for the first time. Any subsequent assessments were excluded from the analysis as that person would no longer be a person without a recent history of falling at that time.*

• You looked at 1 year total time, so some people may have fallen at the end of this period, how did you account for this? You did not really have 1 year followup after a fall (i.e. not long term after a fall). This must be fixed throughout the text and does affect interpretation in discussion.

*We thank the reviewer for this observation. Although we had been careful not to make claims about a one-month or one-year period after a fall, there were several sentences in which this was not done correctly.
We had incorrectly written down the results about the short-term relation between falls and concern for falling in the abstract and have adjusted this as follows:*

Line 30: “Furthermore, falls in between baseline assessment and one year thereafter did not predict increased concern about falling from baseline to one year later, independent of whether all falls or only injurious falls were considered. However, ~~over the course of one month~~, falls, either all or injurious only, happening somewhere over the course of a one-month interval, significantly predicted small increases in concern about falling (1.49 FES-I points, 95% CI [0.74, 2.25], p<0.001 for all falls; 2.60 FES-I points, 95% CI [1.55, 3.64], p<0.001 for injurious falls)from the start to the end of that one-month interval.*”* –

*Note that we, in response to reviewer 3, also added the results from the regression analyses to the abstract]*

*We had also incorrectly summarized our conclusion in the conclusion section of the discussion. We have now corrected this as follows:*

Line 475: “We observed only a small increase in concern about falling ~~within~~ from the start to the end of a one-month interval in people who experienced a fall during this period and ~~a one month period after a fall and~~ observed no increase from the baseline assessment to the end of the study one year later in people who experienced a fall during this period ~~within a one year period after a fall~~.”

*And*

Line 480: “Furthermore, we found no evidence that high concern about falling increases the odds of falling in the following month nor year~~, neither during a one-month nor during a one-year follow-up interval~~.”

*Furthermore, we have added a new figure (fig. 1) that we hope will clarify our study procedures and the conclusions we draw from our data. Please find it included in the uploaded material. The caption that goes along with the figure is included here as well as the reference in the manuscript text:*

**Line 107: “Fig 1. Timeline of study assessments.** Short-term and long-term periods are defined to determine whether concern about falling at the start of such a period is predictive for falling within such a period and whether falling within such a period is predictive of having increased concern about falling at the end of that period compared to the start of that period.. At baseline, a participant’s first assessment, participants reported any falls they had experienced in the previous year and physical activity during one-week, age and gender were recorded.”

**And**

Line 119: “~~Prospective~~ fall~~s~~ incidence

~~Prospective~~ Falls (see definition of a fall under Fall history) were recorded during the long-term study period of one year after baseline assessment (fig. 1) ~~during twelve months~~.”

*To prevent any misunderstandings we also adjusted the method section on statistical analyses:*

Line 201: “~~Does a fall~~Is ~~increase~~ concern about falling increased after a fall?

First, we assessed whether concern about falling was increased after a fall, when comparing the concern about falling at the start of either a short-term (one month) period or the long-term (one year) period with the end of that period~~.over a long-term, i.e. one-year, and short-term, i.e. one-month, interval~~. For the long-term interval, we fitted a linear (fixed-effects) regression model (see equation 1) that predicted changes in concern about falling that occurred between baseline and one- year later ~~follow-up~~, depending on whether people had fallen at least once in between these measurements”

*And*

Line 234: “Does a high concern about falling increase the odds of falling?

Next, we assessed whether the odds of falling was increased with the level of concern about falling, over a long-term, i.e. one-year, and short-term, i.e. one-month, interval. For the long-term interval, we fitted a (fixed-effects) logistic regression model (see equation 3) that predicted the probability of falling during a one-year ~~follow-up~~ depending on participants’ concern about falling at the start of the follow-up period.”

*We also adjusted the results section:*

Line 265: “The development of concern about falling five monthly questionnaires before and five monthly questionnaires after either a non-injurious fall or a injurious fall is displayed ~~over ten months relative to the moment that a fall occurred in people who experienced a fall during the one-year follow-up is displayed~~ in Fig ~~1~~2.”

*And*

Line 285: “~~Does a fall increase~~Is concern about falling increased after a fall?

Over a long-term, i.e. one year , interval, falling (whether or not with an injury) did not significantly change people’s concern about falling from baseline to one-year ~~follow-up~~ after baseline….”

*And*

Line 295: “~~Over a long-term, i.e. one year, follow-up interval, c~~Concern about falling at baseline did not significantly increase or decrease the odds of experiencing a fall during the ~~one-~~year ~~follow-up period~~following the baseline measurement…”

*We also adjusted the discussion section:*

Line 316: “We found that people ~~are~~were more concerned about falling after a fall, injurious or not, but that this increase in concern ~~is~~was was only observable over periods of up to a month and not over periods of up to a year ~~a one-month interval and not over a one-year interval~~.”

• Line 117: By missing the last 3 questions, two of the activities were more likely to be rated of concern for people with a mobility issue (i.e., Walking up or down a slope, Walking on an uneven surface). Also the authors mentioned replacing 4 items so walking in crowds may also be missed. So, just averaging the other may underestimate the score. This should be considered and addressed in limitations.

*The reviewer remarks that the missing FES-I items, due to an error in the electronic version of the questionnaire, may have been items that were more sensitive to detect concern for people with a mobility issue. We agree and have added the following to the discussion:*

Line 445: “Another limitation is that, in 25% of the questionnaires used in this study, three items were accidentally missing and replaced by the mean of the remaining items if no more than four items were missing in total. The missing items were “Walking on an uneven surface”, “Walking up or down a slope” and “Going out to a social event”. To investigate if these missing items influenced the total score of the questionnaires, we compared complete questionnaires and questionnaires with missing items. We did this only for participants who did not experience any falls during the follow-up and who filled in both questionnaires with and without the three missing items during the study duration (N = 34). For each participant we determined the mean score of questionnaires with and without the three missing items and we calculated the difference between these mean scores within all participants. A one-sample t-test showed that the mean difference between the mean questionnaire scores of questionnaires with and without missing items was not significantly different from zero (0.20, 95% CI [-0.24, 0.64], p = 0.367). Although the sample size for this additional analysis was relatively small, it’s results appear to suggest that our method of replacing the missing items was valid.”

• Age, gender, and activity level were used as covariants, but line 145 says missing covariate were replaced with the group mean value. This is inappropriate since you are essentially making up missing data that is specific to the participant. Participants without covariant data should be removed from the analysis.

*We agree with the reviewer that replacing the missing covariates with the group mean value will reduce the amount of variance that can be explained by the covariates and therefore may result in finding an incorrectly stronger association between falls and concern about falling. We have therefore excluded participants with missing covariates (N=3) and updated all results. The update did not affect our conclusions.*

*We have removed the following sentence from the method section:*

Line 168: “~~Missing values for covariates were replaced with the group’s mean value~~”

• Line 151: replace “considered accounting for” with “accounted for” since “considered” implies that you thought about this but did not implement

*The use of the word “considered” reveals to the reader that the confounders are not all included in the model. Instead, they are selected based on two criteria to prevent collinearity and to prevent unnecessary controlling for covariates, which may result in incorrect model coefficients. These criteria are described under the header “statistical analysis”. Based on these criteria, covariates where either entered into the model or not entered into the model. We have included the relevant passage below:*

*Line 195: “*However, these covariates were only added to the models if their correlation with the predictor variable was less or equal to r = 0.6 (to prevent collinearity) and if including the covariate in the model changed the regression coefficient of the main predictor variable (either falling or concern about falling) by 10 percent or more.*”*

• Line 162: It is unclear why regression is used and why predict? You have the data so no need to predict. As well, you indicate the interval is start to end of the year. So this does not involve falls directly, and you cannot day that this is long term 1 year response to falling.

*The aim of our study was to determine whether concern about falling increases after a fall and whether concern about falling increases the odds of future falls in community-dwelling older adults without a recent fall history. We agree with the reviewer that the ideal study design would have an assessment of concern about falling directly before a fall occurred and at several time points (e.g., one month and one year) following the fall. Although the latter would be feasible, it was not how our study was designed. We therefore removed all claims about the response of concern about falling one month or one year after a fall. Instead, we make claims about changes from start to end of one-month and one-year periods when people fell in between these periods, conform our study design. We regret that the mistakes in writing down these claims (in the abstract and conclusion as mentioned in an earlier remark) may have added to the confusion.*

*The reason why we used regression analysis to test our hypotheses is that it fits the aim of our research best. The beta 2 estimates (formula 1 and 2) and the beta 1 estimates (formula 3 and 4) are the increase in FES-I score for people who have experienced a fall and the increase in risk of falling due to FES-I score, respectively. By statistically testing these parameters of our prediction model, determined on the real data that we collected, we have tested our hypotheses.*

*Additionally, in the very first paragraph of the review, the reviewer states the following: “*1-year data was estimated from linear regression”. *We want to clarify that we did not estimate, manufacture or extrapolate data by using regression analysis.*

• Line 176: Again, why regression?

*We hope that our response to the previous comment has clarified why regression analysis is an appropriate method to investigate the aim of our study.*

• Line 193: The authors did not complete a 1 year followup, but tried to predict with an un-validated model. This method is inappropriate, real data is needed to answer the research question

*We hope again that our responses to the previous comments have clarified how regression can be used to test hypotheses about relations between variables and that it can be used to quantify the strength of these relations. We also hope that we have clarified that real data has been used.*

Reviewer #3: I appreciate the opportunity to review this manuscript. This study examined the interrelationship between falling and concern about falling within a short term and a long term follow-up. Results showed that having falls or injurious falls was significantly associated with an increased concern about falling. Overall, the manuscript is well written. There are strengths and weaknesses in this study. My following comments include a summary of weaknesses and offer suggestions for the authors' consideration.

*We thank the reviewer for these kind words and for the much appreciated comments. His/her review has improved the quality of our manuscript*

1. In the Abstract, the methods section should provide the type of statistical analysis. The results section should provide key findings (e.g., beta/odds ratio with 95% CI). The conclusion section should provide implication based on the study results.

*The reviewer remarks that the abstract was missing some vital information. We agree on the remarks about the methods and results section and have added the following:*

*Line 24: “*~~All~~ Standard linear (fixed-effects) regression and mixed effects regression analyses were performed over long-term, i.e. one year, and short-term, i.e. one-month, intervals, respectively and were adjusted for gender, age and physical activity (quantified as the average total walking duration per day).*”*

*And:*

*Line 33: “*However, ~~over the course of one month~~, falls, either all or injurious only, happening somewhere over the course of a one-month interval, significantly predicted small increases in concern about falling (1.49 FES-I points, 95% CI [0.74, 2.25], p<0.001 for all falls; 2.60 FES-I points, 95% CI [1.55, 3.64], p<0.001 for injurious falls)from the start to the end of that one-month interval.*” ---*

*Note that we, in response to reviewer 2 , also corrected the way in which the results were presented in the abstract by more clearly describing that the change in concern happened over one month when a participant had fallen somewhere in this period, as opposed to the other possible interpretation that concern changed one month after the fall.*

*We do not agree about providing (additional) implications based on the study results in the conclusion section. We believe that we already have given a scientific implication in the conclusion section. Namely: “…, suggesting that concern is not a primary cause for falls in older adults without a recent history of falling”.

In the discussion section of the manuscript, we give the following clinical implication: “We cannot advise concern about falling for use in fall prediction models, nor as a target in fall prevention programs aimed at older adults with little concern about falling who have no history of falling.”
We did not include this clinical implication in the abstract’s conclusion section, Not only to keep the abstract concise, but also because the inferred implication is not a direct conclusion of our results.*

2. The Introduction mainly focused on the study by Dalbaere et al about the relationship between falls and fear of falling. The authors should provide a bit more information on how fear of falling affects mobility and activities of daily living, and whether this might have been an underlying mechanism leading to future falls.

*We agree with the reviewer that this information will improve the quality of the manuscript by providing context for our hypothesis. We included the following to the introduction:*

Line 56: “Pathways through which concern about falling may affect the risk of falling have been described previously. Attention allocation while walking is altered in response to concern about falling (5). Threatening objects in the surrounding receive more visual and cognitive attention (6). This adaptation seems to give more time to anticipate obstacles but becomes counterproductive in more complex environments with multiple obstacles, especially while dual-tasking (7, 8), and may lead to trips and falls (9, 10). Furthermore, concern about falling can indirectly lead to falling through activity restriction and thereby induced physical decline (11, 12). It should be noted that it may take time for physical decline to become so severe that it becomes a fall risk.”

*The following references have been added:*

“[5] Gage WH, Sleik RJ, Polych MA, McKenzie NC, Brown LA. The allocation of attention during locomotion is altered by anxiety. Experimental brain research. 2003;150(3):385-94.

[6] Young WR, Mark Williams A. How fear of falling can increase fall-risk in older adults: applying psychological theory to practical observations. Gait Posture. 2015;41(1):7-12.

[7] Ellmers TJ, Cocks AJ, Kal EC, Young WR. Conscious Movement Processing, Fall-Related Anxiety, and the Visuomotor Control of Locomotion in Older Adults. The Journals of Gerontology: Series B. 2020;75(9):1911-20.

[8] Ellmers TJ, Young WR. Conscious motor control impairs attentional processing efficiency during precision stepping. Gait & posture. 2018;63:58-62.

[9] Young WR, Hollands MA. Newly Acquired Fear of Falling Leads to Altered Eye Movement Patterns and Reduced Stepping Safety: A Case Study. PloS one. 2012;7(11):e49765.

[10] Young WR, Wing AM, Hollands MA. Influences of state anxiety on gaze behavior and stepping accuracy in older adults during adaptive locomotion. The journals of gerontology Series B, Psychological sciences and social sciences. 2012;67(1):43-51.

[11] Delbaere K, Crombez G, Vanderstraeten G, Willems T, Cambier D. Fear-related avoidance of activities, falls and physical frailty. A prospective community-based cohort study. Age and ageing. 2004;33(4):368-73.

[12] Hadjistavropoulos T, Delbaere K, Fitzgerald TD. Reconceptualizing the role of fear of falling and balance confidence in fall risk. Journal of aging and health. 2011;23(1):3-23.”

3. One of the inclusion criteria is MMSE score ≥ 19. What is the mean score (SD) of participants? This information should be included in Table 1. Meanwhile, a MMSE score between 19-24 is considered as “mild” cognitive impairment. Are participants with a score within this range able to recall fall history or fill in questionnaires? Please clarify.

*We have added the median MMSE score and interquartile range to Table 1 (MMSE: 28 [28, 29]). The lowest MMSE score in our sample was 24 (n=3). We have added the following to the discussion:*

*Line 463: “*Finally, the lowest MMSE score of participants included in this study was 24 (N=3). An MMSE score between 19 and 24 is generally considered an indication of “mild” cognitive impairment. It may be questioned whether these participants were able to fill out questionnaires and recall falls adequately. However, since there were only three participants with a MMSE score of 24 it is unlikely that this would have affected our conclusions.*”*

4. Covariates in this study included age, gender, and the average walking duration. Were disease diagnoses, MMSE score, and use of mobility aid of participants also available? If so, they should be also included as covariates in the statistical models.

*The reviewer suggests to add additional covariates to the models. The suggested covariates are all available. However, we hesitated to include more covariates because of lack of power. A general rule of thumb regarding predictors in (logistic) regression models is to include 1 predictor per 10 events (Peduzzi and colleagues 1996, DOI:* *10.1016/S0895-4356(96)00236-3). Since there are a total of 40 injurious falls in our study, adding more than 4 predictors is not advised as it may tend to make the results less valid. The aim of this study was to compare the interrelation between falls and concern about falling. Therefore, both sides of relation should be tested with models that were corrected for the same variables and hence also the linear regression models should not be corrected for by more than 4 covariates. However, we have updated Table 1 to describe the presence of diseases, MMSE score and use of mobility aids in our population. We hope that this helps readers to better place our results in context of the population that we have investigated.*

5. This study used an inertial sensor to measure daily activity (e.g., walking duration). How were these data associated with the FES-I scores? In addition, were participants assessed with sensors during the follow-up? It would be interesting to know if having fall(s) influences the physical activity level (e.g., walking duration) objectively measured by the inertial sensors.

*The reviewer remarks that it would be interesting to investigate the relationship between Falls and physical activity and FES-I scores and physical activity. Although we agree that this would be of interest, it is beyond the scope of our manuscript and deserves a separate manuscript dedicated to this new objective.*

6. In this study, nearly half of the participants experienced fall(s) over the one-year follow up. Is there any information about the types of falls? For example, was the fall caused by an internal factor (e.g., balance issue, postural hypotension) or an external factor (e.g., slippery floor, tripping hazard). This is important, as participants who fell due to an internal cause may have higher concern about falling compared to those who fell due to an external cause.

*We agree with the reasoning of the reviewer. Circumstances of the falls were indeed recorded. Participants were asked what they were doing and what caused them to fall. These data may have been influenced by participants concern for falling such that people with poor self-efficacy reported more internal causes, which would potentially have a larger effect on concern about falling. Unfortunately, the obtained qualitative data are not of sufficient quality to assess whether people attributed causes internally or externally. For instance, recordings of causes include: “Participant was cycling, rode into an obstacle and fell.” And “Participant was removing weeds from the garden and lost balance”. We find that these short statements are insufficient for a valid assessment of internal or external attribution of causes.*

7. Among participants who fell, half of them experienced one fall and half experienced two or more falls. The analysis only examined the association between falling and concern about falling. The authors may want to investigate whether people having more falls would have a higher concern about falling than those having one fall.

*The aim of the research presented in our manuscript was to study the reciprocal relation between falls and concern about falling. We compare the two directions of this relation over short and long-term intervals. The short-term interval is the most innovative part of our research. Comparison between short-term and long-term intervals, which is an important part of our study, would not be possible when we analyze people with two or more falls separately from people with one fall. This is because there are very few people who fell twice in one month and therefore the short-term (mixed effects) regression models would have to low a sample size. However, severity of falls is instead expressed by analyzing injurious and all falls separately.*

8. In the discussion, the authors may want to highlight the importance of understanding the interrelationship of falling and concern about falling, and provide implications/ recommendations based on the study findings.

*We have added the following to the discussion section:*

Line 468: “Our findings imply that falls are not the primary cause of sustained concern about falling in older adults without a recent history of falling. Although concern about falling is increased up to one month after a fall, this effect was not seen after a period of up to a year. Clinicians and other healthcare professionals should be aware that there may be other factors besides recent falls, even those that result in injuries, that may cause older adults to become concerned about falling or that result in a sustained level of concern about falling”

9. The authors stated that “a strength of the study is the exclusion of people with a self-reported history of falling”. However, the rate of falling in this study is quite high (nearly 50%) compared to the falling rate in community-dwelling older adults (about 30% in general). It is surprising to see that none of these participants had a fall history at baseline. This suggests the need to investigate what caused them to fall in this study (see comment #6). Please provide explanations.

*Based on Rubenstein and colleagues 2006, DOI: 10.1093/ageing/afl084, the falling rate in community dwelling older adults, independent of recent history of falling, is somewhere between 30% and 60%. Fall history has been found to be associated with future falls and therefore a lower fall rate may be expected in people who reported no recent fall history. However, further analyses of our data also showed that fall history may be prone to recall bias associated with concern about falling. Possible explanations for the high number of falls in our population may be the rigorous follow-up for falls and the broad definition of a fall used in our study. Based on the study by Rubenstein and colleagues 2006 and our finding that recall bias may underestimate the number of falls when falls are assessed retrospectively, we believe that the number of falls in our population is normal. Regarding the recall bias related to retrospectively assessing falls, we have added the following to the discussion:*

*Line 377: "*Moreover, to evaluate the association between recent fall history and concern about falling, we performed linear regression analyses similarly to the long-term, i.e., one-year, models described in the method section. In one model recent fall history predicted concern about falling at baseline and we compared its results with a second model in which falls recorded during the one-year monthly follow-up predicted concern about falling at the end of that follow-up (not adjusted for concern about falling at baseline). The results and comparison of these results indicated that history of falling was predictive of high concern about falling at baseline (βfall= 1.79, 95% CI [0.64 , 2.93], points on the FES-I, p = 0.002), whereas number of falls during the one-year follow-up was not predictive of concern about falling one year after baseline (βfall= 1.05, 95% CI [-0.28, 2.38], points on the FES-I, p = 0.123) and (βinjuriousfall= 0.86, 95% CI [-0.53, 2.25], points on the FES-I, p = 0.225). Recall bias may be an explanation for this finding, as recall bias is likely to be more pronounced when the recall period is large, i.e., when asking about recent fall history retrospectively instead of assessing falls prospectively using a monthly follow-up. Concern about falling may possibly lead to recall bias, such that people with high concern about falling recall more falls than people with low concern about falling. This recall bias stresses the need for prospective fall assessments using monthly telephone contact or fall diaries, such as we employed in our study, to validly record falls in older adults in future studies.

*And we have removed the following from the discussion as it was redundant after adding the previous section:*

*Line 423: ~~“~~*~~we believe that such discrepancies may further support our belief that self-reported history of falling may either bias findings concerning the relationship between falls and concern about falling, or that this relationship is different for people with or without a recent history of falling.~~”

*In addition to the reviewers comments we found typos and changed them:*

Line 319: “We found no evidence that concern about falling predicts falls, neither over a one-month interval ~~not~~ nor over a one-year interval.

Line 64: “Delbaere and colleagues (13, 14) recorded falls every month and concern about falling, measured with the FES-I, every three months in a group of older adults (70-90 years o~~i~~f age), some of whom had a history of falling.”

*We also adjusted a sentence for better flow of the manuscript:*

Line 460: “Furthermore, in our population we mostly recorded minor injuries after a fall (e.g. bruises and small cuts). This could be considered a limitation as~~, whereas~~ falls resulting in more severe injuries could may have a larger impact on concern about falling.”

*We also removed the funding statement from the acknowledgements section to comply with PLOS ONE standards.*

Line 487: “~~This research was funded by a VIDI grant (no. 91714344) from the Dutch Organization for Scientific Research (NWO).~~”

*Finally, we added the sample size per month to figure 2 and adjusted the description of the figure.*

*Line 276: “*Fig ~~1~~2. Progression of concern about falling before and after a fall. Distribution of FES-I scores per month are displayed in dark grey relative to the months in between which the participant experienced his or her first fall (area indicated in light gray) (-1 = last monthly questionnaire before the fall, +1 = first monthly questionnaire after the fall). Diamonds and solid line represent the mean FES-I scores and the progression of these mean FES-I scores, respectively. The dark grey areas represent the distribution of FES-I scores per month. The upper figure displays FES-I progression before and after any type of fall. The lower figure displays FES-I progression before and after an injurious fall. FES-I scores of people who fell more than once are only included until the first monthly questionnaire after the first fall~~month before the second fall~~. Sample size (N) is indicated for each month.”

6. PLOS authors have the option to publish the peer review history of their article ([what does this mean?](https://journals.plos.org/plosone/s/editorial-and-peer-review-process#loc-peer-review-history)). If published, this will include your full peer review and any attached files.


If you choose “no”, your identity will remain anonymous but your review may still be made public.


**Do you want your identity to be public for this peer review?** For information about this choice, including consent withdrawal, please see our [Privacy Policy](https://www.plos.org/privacy-policy).

Reviewer #1: No

Reviewer #2: No

Reviewer #3: No
